# Development of a questionnaire to evaluate practitioners’ confidence and knowledge in primary care in managing chronic kidney disease

**DOI:** 10.1186/1471-2369-15-73

**Published:** 2014-05-07

**Authors:** Mohammad Tahir, Simon Hassan, Simon de Lusignan, Lazza Shaheen, Tom Chan, Olga Dmitrieva

**Affiliations:** 1 Department of Health Care Management and Policy, School of Management, University of Surrey, Guildford, Surrey GU2 7XH, UK; 2 AT Medics, Edith Cavell Surgery, 41A-C Streatham High, London SW2 4TP, UK

**Keywords:** CKD, Confidence, Kidney Failure, Chronic, Blood pressure, Primary Care, Questionnaire, Quality of Healthcare

## Abstract

**Background:**

In the UK, chronic disease, including chronic kidney disease (CKD) is largely managed in primary care. We developed a tool to assess practitioner confidence and knowledge in managing CKD compared to other chronic diseases. This questionnaire was part of a cluster randomised quality improvement interventions in chronic kidney disease (QICKD; ISRCTN56023731).

**Methods:**

The questionnaire was developed by family physicians, primary care nurses, academics and renal specialists. We conducted three focus groups (n = 7, 6, and 8) to refine the questionnaire using groups of general practitioners, practice nurses and trainees in general practice. We used paper based versions to develop the questionnaire and online surveys to test it. Practitioners in a group of volunteer, trial practices received the questionnaire twice. We measured its reliability using Cohen’s Kappa (*K*).

**Results:**

The practitioners in the focus groups reached a consensus as to the key elements to include in the instrument. We achieved a 73.1% (n = 57/78) initial response rate for our questionnaire; of these 57, 54 completed the questionnaire a second time. Family physicians made up the largest single group of respondents (47.4%, n = 27). Initial response showed more female (64.9%, n = 37) than male (35.1%, n = 20) respondents. The reliability results from retesting showed that there was moderate agreement (*k* > 0.4) on all questions; with many showing substantial agreement (*k* > 0.6). There was substantial agreement in the questions about loop diuretics (*k* = 0.608, CI 0.432-0.784, p < 0.001), confidence in managing hypertension (*k* = 0.628, 95%CI 0.452-0.804, p < 0.001), diastolic blood pressure treatment thresholds in CKD (*k* = 0.608, 95%CI 0.436-0.780, p < 0.001) and the rate of decline of eGFR that would prompt referral (*k* = 0.764, 95%CI 0.603-0.925, p < 0.001).

**Conclusion:**

The QICKD-CCQ is a reliable instrument for measuring confidence and knowledge among primary care practitioners on CKD management in the context of UK primary care.

## Background

The management of chronic kidney disease (CKD) is a new challenge for primary care practitioners [1,2]. It involves the risk stratification of patients by disease severity using primary care data [3-5]. CKD has been included in the range of guidelines published by the National Institute for Health and Clinical Excellence (NICE) since 2008, primary care clinicians are expected to implement these [6]. These guidelines follow those of The National Kidney Foundation Kidney Disease Outcomes Quality Initiative (NKF KDOQI™) [7]. Additionally, in the UK, CKD management has been added to pay-for-performance (P4P) targets for primary care. P4P was introduced for chronic disease management in primary care in 2004, and extended to include CKD in 2006 [8]. Whilst the response to P4P has been mixed [9-11], it is plausible that these P4P interventions might impact on renal replacement therapy, as P4P represents a form of quality improvement which therefore could slow disease progression in CKD [12,13]. When CKD was added to the quality indicators, little was known about how to improve quality in this condition [12]. The Quality Improvement in CKD trial (QICKD) [14] included a systematic review [12] and diagnostic analysis [15] to explore factors limiting achievement of quality improvement in CKD. This revealed that primary care practitioners had gaps in their knowledge, highly variable views about this condition and lacked confidence in explaining and managing the condition [15,16].

We are not aware of any questionnaires to measure practitioner confidence and knowledge in the management of CKD. Despite this, clear guidance does exist on priority areas to address in patients with CKD, such as systolic blood pressure and proteinuria [6,17,18]. Questionnaires provide a reliable way of measuring confidence and knowledge if properly developed. We developed this questionnaire as part of the process of evaluating of a large cluster randomised trial: the QICKD trial [19]. The QICKD has three arms: “Audit based education” (feedback of performance compared with peers in an educational context) [20]; “Guidelines and prompts” (postal reminders about management of CKD and copies of national guidance) [21]; and usual practice. This paper reports how we developed and tested this questionnaire to provide a reliable instrument to measure confidence and knowledge in managing CKD. We would like this questionnaire to be used as a tool to measure confidence and knowledge in managing CKD with an emphasis on managing blood pressure control in these patients.

## Method

### Literature review

We carried out a literature review [12], a diagnostic analysis [15] and these were summarised in the overall protocol [19] to identify whether there were any existing validated tools we could use for measuring confidence in CKD. We particularly looked for tools developed for the management of other cardiovascular diseases including diabetes, as strict management of BP and of proteinuria are key aspects of the primary care management of both conditions. We also searched for other literature about the validation of questionnaires to discover the typical sample sizes used and expected response rate from test-retest studies [22,23].

### Developing a valid questionnaire

We agreed the key objective for the questionnaire was to find out how confident and knowledgeable practitioners were in managing the QICKD’s primary outcome measure: systolic blood pressure (SBP). As we could not make absolute measures of confidence we decided to compare confidence levels against other well established chronic conditions managed in primary care. These chronic conditions were felt to be important and comparable by our project group which consisted of general practitioners, nurses and renal specialist. The questionnaire content was further tested with the focus groups below.

The final objectives of our questionnaire were:

• To compare the confidence of practitioners in controlling systolic BP in patients with CKD with that in patients with hypertension alone. Improved control of systolic BP is the primary outcome measure of the QICKD [12].

• To compare confidence in managing proteinuria in CKD with diabetes. Patients with CKD and proteinuria are at high risk of adverse renal and cardiovascular outcomes [17].

• To compare confidence levels in General Practitioner (GP) partners, salaried GPs and nurses. Our systematic review indicated that successful initiatives in CKD had often been non-doctor led [12].

• To create a questionnaire that a busy primary care practitioner, GP or nurse, could complete in a maximum of 10 minutes.

• The focus groups also addressed the importance of including confidence in initiating antihypertensive therapy angiotensin converting enzyme inhibitors (ACE-I) and angiotensin II receptor blockers (ARB) according to guidelines.

The knowledge questions were about BP targets for treatment, intervention levels of proteinuria and criteria for referral. The questions asked for numeric responses with respect to: BP management; change in renal function requiring referral (measured with an estimate of glomerular filtration rate (eGFR)); quantitative measures of proteinuria (in the UK measured using albumin creatinine ratio (ACR)); and decline in renal function that should result in referral to specialist care.

We used an established four stage method to develop the confidence questionnaire: (1) Planning, (2) Piloting, including response formats, (3) Layout finalisation and question ordering, (4) Developing the covering letter/distribution method [24]:

1. Planning

We developed a project plan for the development of the questionnaires and appointed a project team from within the QICKD study team. We developed further objectives based on our study using knowledge gained from our systematic review^12^ and diagnostic analysis [15]. We planned to distribute the questionnaire initially on paper, with the option of follow-up questionnaires being completed online. The additional areas in the project plan concerned the resources required to complete the questionnaire.

2. Piloting

We circulated a draft questionnaire amongst the investigators. We then held a group discussion to identify current issues in CKD management, to check the objectives developed in the planning phase, and identify any new items. At these meetings, we elected to include a small number of key knowledge questions, as it was felt that confidence could not be interpreted in complete isolation. We defined the ‘confidence’ components as what individuals know about their ability–in this case based on the knowledge of guidelines, as well as previous experience of the task. We selected components in our questionnaire that we thought would be appropriate surrogates to a broader level of components. We used a ranking exercise to prioritise the areas of knowledge to be tested. We decided to restrict our knowledge test to key data that had numeric responses. This draft questionnaire was then piloted on practitioners. This cycle was repeated on three occasions. We conducted three focus groups (n = 7, 6, and 8) to refine the questionnaire using groups of general practitioners, practice nurses and trainees in general practice. These focus groups were facilitated by AT, and attendees were asked to comment on what they thought should be the primary focus of the questionnaires. The process involved the practitioners completing the questionnaire and then marking a colleague’s questionnaire while discussing the responses and highlighting potential ambiguities. We chose to “mark” each other so that we could encourage an open discussion about the responses given by our colleagues. The focus groups’ comments were captured using a field notebook, the annotated questionnaires, flipchart notes capturing the key findings and tape recording of two out of three of the focus groups (one recording failed), which were subsequently transcribed. A smaller group of GPs was contacted via email (n = 3) to test the feasibility of collecting data remotely [25]. We reviewed our findings and found that we had significantly increased the number of questions during this process, and that we needed to subsequently reduce them. We felt we needed to keep the number to a manageable time limit of around 5–10 minutes as this felt acceptable to the participants and more likely to return a greater response rate.

3. Layout finalisation and question ordering

We explored various layouts and arrangements of the questionnaire with our focus group attendees and then asked them to test the final version online. Focus groups also suggested that we should give the questionnaire a logical progression, mimicking the traditional progression in medical management of a condition. We therefore initially grouped questions about history, investigation and diagnosis. The focus groups found our initial ordering difficult with some participants “flipping” the Likert scale. In other words participants felt that ‘5’ should represent very confident and ‘1’ not confident at all. We therefore accepted their recommendations, and we added shading and “smiley faces” ranging from confident (happy) through to not-confident (sad). The final questionnaire consisted of 24 confidence questions and 6 knowledge questions (Additional file 1). The confidence questions used a five item Likert scale, where 1 is “Not confident at all” and 5 is “Very confident.”

4. Developing the distribution method including the covering letter

Each clinician received an email link to complete the questionnaire (Survey Monkey Questionnaire™). We prompted non-responders up to three times for each collection. We used a combination of email announcing the intention to telephone, followed up by a phone call if there was no response. The process was repeated, allowing a minimum of two and no longer than five weeks between the test and retest; to test rater-rater reliability. The participants were not compensated for the time taken to do the questionnaire.

### Sampling frame for testing questionnaire reliability

We identified a sample of 78 practitioners, drawn from a network of 13 practices: either in-depth process evaluation practices or others were associated with the principal and a senior researcher to test the reliability of the questionnaire. The geographical spread of these practices is in southern England and represent practices with a registered list size ranging from 2600 up to 12000 patients. The in-depth process evaluation practices were a group of practices in the QICKD trial which received the same intervention as the main trial practices but were also observed so we could learn about programme fidelity (i.e. the extent to which the intervention ran as planned) and intervention exposure (i.e. the extent to which members of trial practices were exposed to the intervention) [19,15]. We aimed to achieve 50 paired responses. Each clinician was required to complete the questionnaire on two separate occasions between 2-5 weeks apart. We included paired responses and considered a valid response to be a questionnaire with more than 80% of the responses included. Paired responses are required to calculate a Cohen’s kappa. Paired questionnaires were only requested from those initially completing the questionnaire on the first collection sample. The investigators blinded themselves from the data collection and analysis; this was through the appointment of a researcher to administer the questionnaire delivery. Any surveys completed by telephone or on paper were inputted by a research assistant.

### Statistical methods for testing reliability

Rater-rater reliability is a measure used to examine the agreement between two ratings on the assignment of categories of a categorical variable. It is an important measure in determining how well the implementation of a coding or measurement system works. We used Cohen’s Kappa (*K*) as a statistical measure of rater-rater reliability. The ranges of this coefficient are generally between 0 to 1.0 (although negative numbers are possible), where large numbers mean better reliability and values near or less than zero suggest that agreement is attributable to chance alone [26]. We categorised the level of agreement achieved using Cohen’s Kappa as follows: Less than zero: poor agreement; 0.0-0.20: slight agreement; 0.21-0.40: fair agreement; 0.41-0.60: moderate agreement; 0.61-0.80: substantial agreement; 0.81-1.00: almost perfect agreement. Where there was asymmetry between test and retest answers, we collapsed the categories where there was no response so that we could make a valid comparison.

### Ethical considerations

This questionnaire development does not require ethical approval, however the questionnaire will be used as part of the QI-CKD study which was approved by the National Research Ethics Service’s Oxford Research Ethics committee and is a registered clinical trial (ISRCTN56023731).

## Results

### Reliability testing

**Figure 1:**
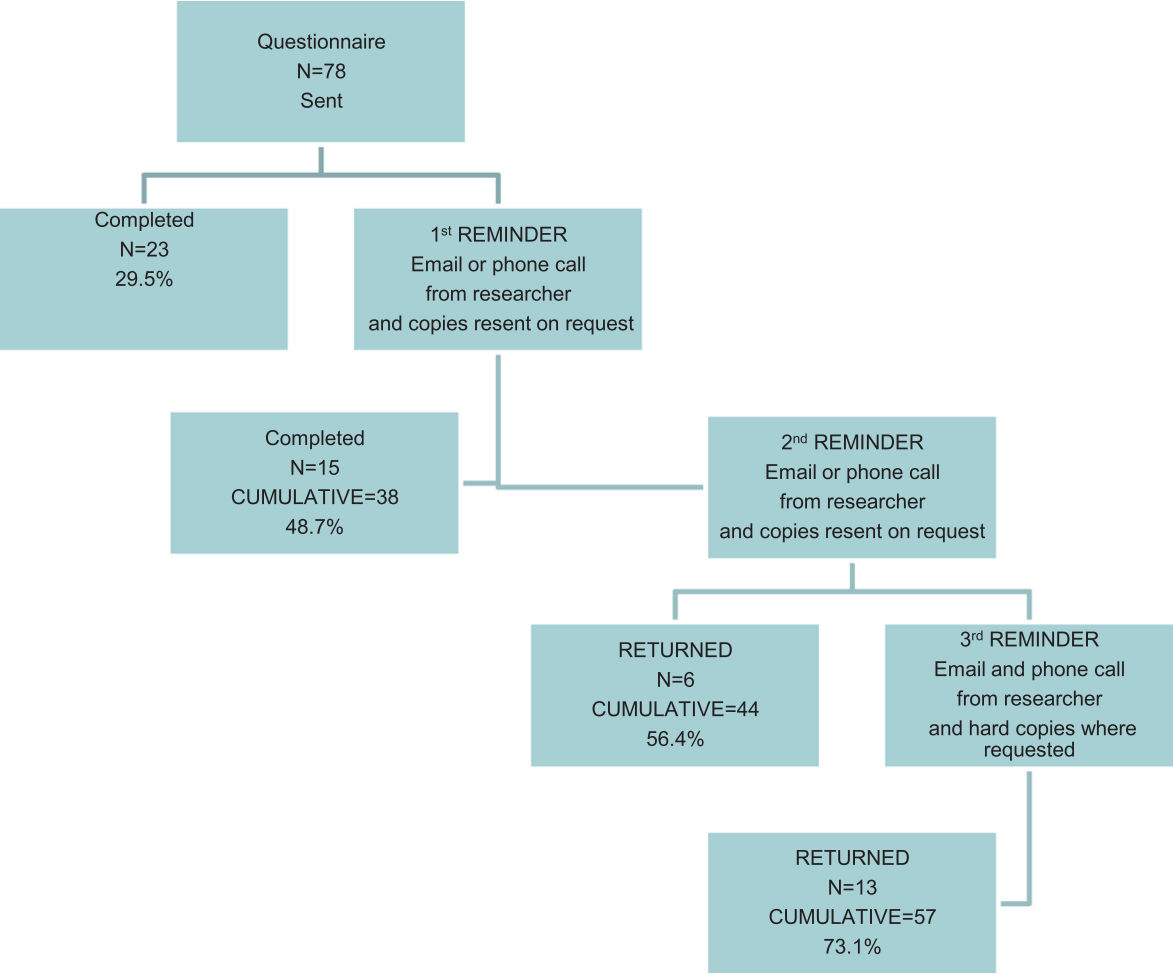
First response to distribution of questionnaires for testing reliability.

**Figure 2:**
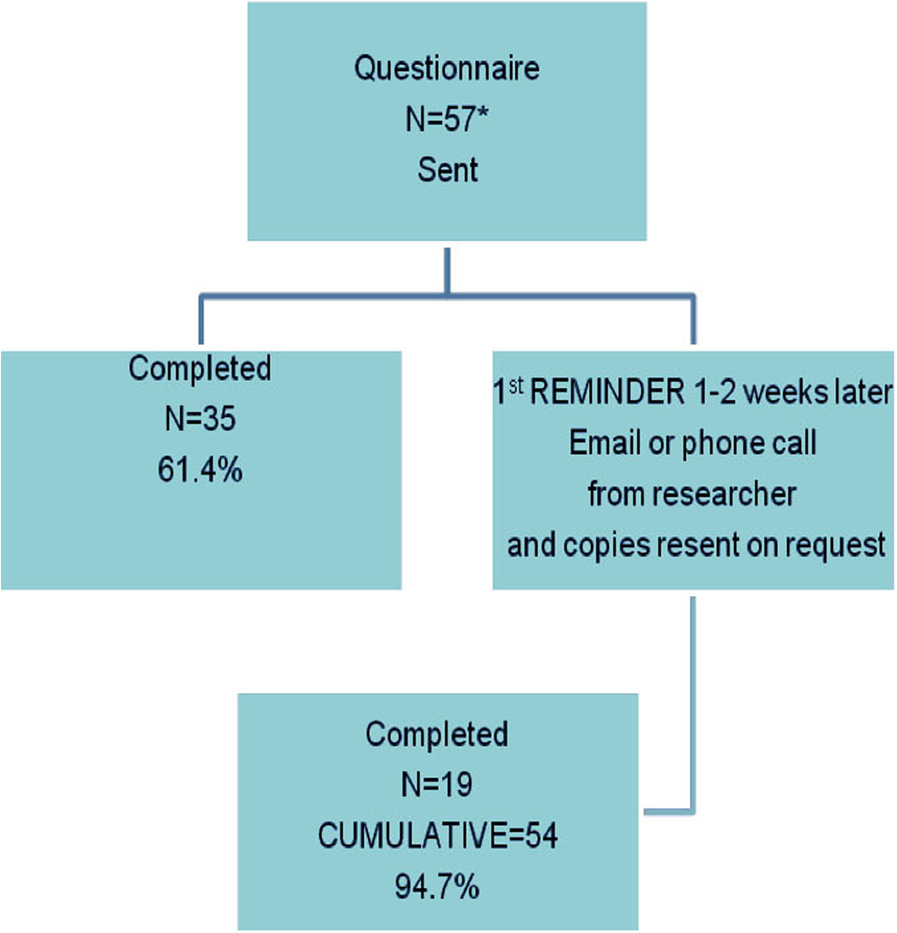
Testing of second round of questionnaires for reliability.

The response rate for the first questionnaire was 73.1% (n = 57/78) valid questionnaires. There were no invalid responses. Of the 57 primary care professionals (PCP) who completed the first round 54 completed the second: a 69.2% (n = 54/78) response rate providing 54 paired questionnaires. Responses were received from all 13 practices taking part (Figure 1, Figure 2). We had 54 paired responses; however one 2nd round response had incomplete data. We therefore were unable to compare this data for the responses to the majority of the questions but we were able to identify the characteristics of the respondent. Therefore for Cohen’s kappa calculations we used 53 paired responses.

General practitioners who are partners and salaried GP’s made up the largest single group of respondents (47.4%, n = 27) followed by nurses (28.1%, n = 16). Locums and trainees made up the smallest group of respondents (Table 1).

**Table 1:** Role and response rates

**Roles**	**Absolute sent out**	**Completed Round 1**	**Completed both Rounds**
GP	41	27 (47.4%)	25 (46.3%)
Locum GP	8	8 (14.0%)	8 (14.8%)
Trainee GP	6	6 (10.5%)	6 (11.1%)
Nurse	23	16 (28.1%)	15 (27.8%)
Total	78	57 (73.1% of sent out)	54 (69.2% of sent out)

There were more female respondents (64.9%, n = 37) than male (35.1%, n = 20) (Table 2). More of the female respondents of this sample are in the older age bands and all the nurses are female (28.1%, n = 16). There was an equal split between practitioners who work full and part-time (49.1% vs. 50.9%). More female clinicians work part-time than male clinicians. However, this finding is not statistically significant.

**Table 2:** Demographic status and response rate of the participants

**Gender**	**Age Band**				**Employment Status**		**Total**
	**25-34**	**35-44**	**45-54**	**55-64**	**Full-time**	**Part-time**	
Male	10	9	1	0	12	8	20
Female	12	7	13	5	17	20	37
Total	22 (38.6%)	16 (28.1%)	14 (24.6%)	5 (8.8%)	29 (50.9%)	28 (49.1%)	57 (100%)

There were 53 valid pairs of questionnaires we could calculate a Kappa coefficient for, with all but two questions having symmetry in the result. All the Kappa coefficients for the confidence questions are within the moderate to substantial range of agreement (*K* > 0.4) and were statistically significant. There was substantial agreement in the questions about loop diuretics (k = 0.608, 95% CI 0.432-0.784, p < 0.001,) and confidence in managing hypertension (k = 0.628, 95% CI 0.452-0.804, p < 0.001,). Two pairs of ratings were asymmetrical, for example the question about confidence with hypertension. This pair of ratings was re-coded to three categories and Kappa statistics calculated (Table 3).

**Table 3:** Cohen’s Kappa showing rater-rater reliability for confidence questions

**Question stem–*How confident are you at:***	**Cohen’s Kappa**	**SE**	**Probability p**	**95% CI**	**Count n**
…managing hypertension as a disease?	0.458	0.113	p < 0.001	0.237 to 0.679	53
…managing hypertension in patients with CKD?	0.628	0.090	p < 0.001	0.452 to 0.804	53
…managing hypertension in patients with CKD with Diabetes?	0.562	0.095	p < 0.001	0.376 to 0.748	53
..that you can achieve lowered blood pressure in patients with CKD?	0.569	0.103	p < 0.001	0.367 to 0.771	53
…interpreting eGFR to stage CKD?	0.561	0.087	p < 0.001	0.39 to 0.732	53
..with monitoring eGFR in patients with CKD?	0.426	0.092	p < 0.001	0.246 to 0.606	53
…monitoring eGFR in CKD patient with Diabetes?	0.413	0.091	p < 0.001	0.235 to 0.591	53
…identifying significant proteinuria in patients with Diabetes?	0.495	0.097	p < 0.001	0.305 to 0.685	53
…identifying significant proteinuria in patients with CKD?	0.481	0.094	p < 0.001	0.297 to 0.665	52
…using urine protein (ACR or PCR) results to manage Diabetes?	0.421	0.093	p < 0.001	0.239 to 0.603	52
…using urine protein (ACR or PCR) results to manage CKD?	0.486	0.094	p < 0.001	0.302 to 0.67	51
…using ACE inhibitors and / or ARB’s?	0.589	0.094	p < 0.001	0.405 to 0.773	53
…using ACE inhibitors and / or ARB’s in patients with CKD?	0.591	0.089	p < 0.001	0.417 to 0.765	53
..using other anti-hypertensives in patients with CKD?	0.508	0.096	p < 0.001	0.32 to 0.696	53
…in adding a loop diuretic drug to patients with CKD (stage 3b and above) already on maximum dose of an ACE inhibitor and/or ARB?	0.608	0.0896	p < 0.001	0.432 to 0.784	53
…identifying CVD risk factors for patients with CKD?	0.573	0.100	p < 0.001	0.377 to 0.769	53
…assessing CVD risk scores in patients with Diabetes?	0.539	0.097	p < 0.001	0.349 to 0.729	53
…assessing CVD risk scores in patients with CKD?	0.523	0.099	p < 0.001	0.329 to 0.717	53
…initiating therapy to lower lipid levels in patients with heart disease?	0.557	0.106	p < 0.001	0.349 to 0.765	53
…initiating therapy to lower lipid levels in patients with CKD?	0.603	0.091	p < 0.001	0.425 to 0.781	53
…using referral guidelines to refer appropriate patients with Diabetes (Type 2) to secondary care?	0.489	0.095	p < 0.001	0.303 to 0.675	53
…using referral guidelines to refer appropriate patients with CKD to secondary care?	0.558	0.086	p < 0.001	0.389 to 0.727	53
…the overall management of patients with Diabetes (Type 2)?	0.545	0.098	p < 0.001	0.353 to 0.737	53
…the overall management of patients with CKD?	0.543	0.091	p < 0.001	0.365 to 0.721	53

Our knowledge questions showed Kappa coefficients within the moderate to substantial range of agreement and all were statistically significant. There was substantial agreement for the questions about diastolic blood pressure treatment in CKD without proteinuria (k = 0.608, 95% CI 0.436-0.780, p < 0.001,) and the rate of decline of eGFR that would prompt referral to secondary care (k = 0.764, 95% CI 0.603-0.925, p < 0.001,) (Table 4).

**Table 4:** Cohen’s Kappa showing rater-rater reliability for knowledge questions

**Question–Stem: *What level of…***	**Kappa**	**SE**	**p**	**95% CI**	**n**
At what level of eGFR would you typically refer to secondary care?	0.551	0.098	p < 0.001	0.359 to 0.743	53
… SYSTOLIC blood pressure control do you typically aim to achieve in patients with CKD WITHOUT proteinuria?	0.472	0.123	p < 0.001	0.230 to 0.713	53
… DIASTOLIC blood pressure control do you typically aim to achieve in patients with CKD WITHOUT proteinuria?	0.608	0.088	p < 0.001	0.436 to 0.780	53
… SYSTOLIC blood pressure control do you typically aim to achieve in patients with CKD WITH proteinuria?	0.583	0.099	p < 0.001	0.389 to 0.777	53
… DIASTOLIC blood pressure control do you typically aim to achieve in patients with CKD WITH proteinuria?	0.482	0.100	p < 0.001	0.286 to 0.678	53
What rate of decline per annum in eGFR would prompt you to refer to secondary care?	0.764	0.082	p < 0.001	0.603 to 0.925	53

We attach a correlation matrix as an additional means to assess how similar or dissimilar one question is to another (Additional file 2).

## Discussion

### Principal findings

The Clinician Confidence and Knowledge Questionnaire (CCQ) appears to be a reliable instrument in testing confidence and knowledge in the management of CKD, hypertension and diabetes. There was consensus about which items should be included in such a questionnaire, and about the key areas of knowledge. The CCQ provides higher levels of reliability when testing knowledge rather than confidence. The CCQ appears easy to complete with nearly all volunteers completing the paired questionnaires.

### Implications of the findings

The appropriateness of the questionnaire is framed by the UK context and guidelines at the time the instrument was created, though these do not vary substantially from international trends in managing this condition. However, whilst the knowledge questions may change over time, comparing confidence with the management of other chronic diseases in primary care is likely to remain a valid, albeit changing, comparison. The CCQ enables a reliable measure of knowledge and confidence to be assessed. It can be used as a tool to assess interventions that might improve the confidence and knowledge of primary care practitioners. If we accept the link between confidence and knowledge this might enable a measure of confidence as a proxy for improving knowledge.

### Comparison with the literature

Studies have shown a knowledge gap in CKD management [27-29], reinforcing the findings of our own diagnostic analysis [15]. Further, a recent study of the use of laboratory prompts shows that they alone are not sufficient to raise the standard of care [30]. However, there are some pointers from educational research and from other disease areas that level of knowledge [31,32] and confidence is associated with improved practice; however most of these articles are descriptive rather than trial-based. A literature search on questionnaires and reliability revealed very few articles associated with confidence; though a city-based collaborative which also included education and better use of technology appears to have improved care [33].

### Limitations of the study

We received less than 80% for the initial response rate (n = 57, 73.1%), however our paired response rate was 94.7% (n = 54/57). There may be a number of sources of bias in this investigation. Firstly, the practices participating in this study are pre-selected by the researchers and may not be representative of the wider population and we do not report on non-responders. The study does not compare confidence and knowledge to clinical outcomes for patients, namely, their enablement [34] or awareness of their diagnosis [35], and so cannot associate high scores with improved care. We also do not measure competence because competence includes knowledge, skills and attitudes [36]. It is possible that treatment thresholds or the measures of renal function or for testing proteinuria may vary between health systems; however these aspects of the questionnaire can be adapted to keep abreast of a changing evidence base and practice. For example, the use of total protein creatinine ratio rather than albumin and creatinine ratio (ACR) as a proteinuria test; or the use of another method of measuring renal function.

### Call for further research

We need to compare confidence and knowledge with clinical outcomes in CKD [31]. Further studies of the provision of educational or other interventions to people who lack confidence in managing a condition should be conducted to determine whether this improves the quality of care.

## Conclusion

The CCQ instrument should be added to the armamentarium of improvement tools and confidence measures in primary care practitioners for the assessment of confidence in chronic kidney disease, hypertension and diabetes.

### Questionnaire available from

http://www.clininf.eu/qickd-ccq.html.

## Abbreviations

ACR: Albumin creatinine ratio; ACE-I: Angiotensin converting enzyme inhibitors; ARB: Angiotensin II receptor blockers; BP: Blood pressure; CKD: Chronic kidney disease; CCQ: Clinician Confidence and Knowledge Questionnaire; eGFR: Estimate of glomerular filtration rate; GP: General Practitioner; NICE: National Institute for Health and Clinical Excellence; NKF KDOQI: National Kidney Foundation Kidney Disease Outcomes Quality Initiative; P4P: Pay-for-performance; PCP: Primary care professionals; QICKD: Quality Improvement in Chronic Kidney Disease.

## Competing interests

AT is a director of AT Medics, delivering care to over 80,000 patients in 16 practices across London.

SH is a GP working in AT Medics.

SdeL Is the GP expert advisor for the Quality and Outcomes Framework (QOF–pay-for-performance (P4P scheme) with the role of developing a CKD Indicator. This was done on behalf of NICE (National Institute for Health and Clinical Excellence). SdeL has received funding for research staff from Roche for the data analysis which formed part of the NEOERICA study. He has received sponsorship from Pfizer to speak at two cardiovascular meetings in 2008; received an honorarium for writing a magazine article (Prescriber).

LS Is a clinical administrator with AT Medics and a research assistant.

TC Nil declared.

OD Nil declared.

## Authors’ contributions

AT: Developed and validated confidence questionnaire, contributed to analysis of data, major part in drafting and redrafting paper, contributed to each version of the paper. SH: Contributed to the final versions of the paper. SdeL: Developed the idea of assessing confidence and knowledge, contributed to analysis of data, contributed to each version of the paper, PI of QICKD study. LS: Collection of data, development of online instrument, contributed to final version of paper.TC: Contributed to developing questionnaire, analysis of data, contributed to final version of paper. OD: Contributed to final version of paper. All authors declare that the answers to the questions on your competing interest form are all “No”. All authors have read and approved the final manuscript.

## Pre-publication history

The pre-publication history for this paper can be accessed here:

http://www.biomedcentral.com/1471-2369/15/73/prepub

## Supplementary Material

Additional file 1 Questionnaire: Evaluating Primary Care Practitioners’ confidence and knowledge in managing chronic kidney disease. Click here for file

Additional file 2 Correlation Matrix. Click here for file
